# Current Scenarios and Future Perspectives of Therapeutic Drug Monitoring in India: A Narrative Review

**DOI:** 10.7759/cureus.102678

**Published:** 2026-01-30

**Authors:** Joyeta Paul, Prasanjit Das, Alapan Das, Bisweswar Ojha, Krishnasish Das, Tirthankar Deb

**Affiliations:** 1 Biotechnology, Maulana Abul Kalam Azad University of Technology, Haringhata, IND; 2 Pharmacology, All India Institute of Medical Sciences, Kalyani, Kalyani, IND; 3 Pharmacology, ESI-PGIMSR, ESIC Medical College and Hospital, Kolkata, IND

**Keywords:** eastern india, high-performance liquid chromatography (hplc), lcms, point-of care testing, therapeutic drug monitoring (tdm)

## Abstract

Therapeutic drug monitoring (TDM) is a precision medicine tool that measures drug concentrations in biological fluids to guide individualized dosing. TDM has evolved into a multidisciplinary approach used across various therapeutic areas. In India, despite a growing clinical need, TDM remains underutilized due to systemic and infrastructural barriers. This narrative review aims to summarize the evolution, current status, analytical approaches, and future prospects of TDM in India and is based on published literature retrieved from databases, including PubMed, Scopus, and Google Scholar, and outlines TDM's utility in optimizing pharmacotherapy for drugs with narrow therapeutic windows or nonlinear pharmacokinetics. It compares analytical methods used for TDM and discusses challenges in the workflow of TDM. Special emphasis is placed on its relevance in children, pregnant women, the elderly, and critically ill patients. It also highlights barriers, including a lack of infrastructure, trained personnel, and standardized guidelines. TDM in India requires policy-level support, integration into national health programs, clinician education, and adoption of point-of-care technologies. Strengthening research in pharmacogenetics can help establish TDM as a cornerstone of individualized therapy.

## Introduction and background

Therapeutic drug monitoring (TDM) is referred to as the individualisation of dosage by maintaining plasma or blood drug concentrations within a target range (therapeutic range, therapeutic window) [1]. It ensures clinical benefit while decreasing the risk of toxicity. This is crucial for medications with narrow therapeutic indices, where even a minor dosage variation can differentiate between effective treatment, therapeutic failure, and serious toxicity. Moreover, it is indispensable for drugs with nonlinear pharmacokinetics. TDM reinforces the idea that "one size does not fit all" and supports drug dosing based on individual characteristics. Hence, it can help modify dosages of the medications considering the patient's age, weight, organ function, etc. [2]. In the past, TDM services were first offered by institutions such as King Edward Memorial Hospital in Mumbai in the late 1980s. Christian Medical College, Vellore, and Postgraduate Institute of Medical Education and Research, Chandigarh, were the next to follow [3]. During the early 2000s, the opportunity of TDM extended beyond its early focus on antiepileptic drugs, including monitoring of several other drug categories such as mood stabilizers, anti-cancer drugs, and cardiac glycosides where maintaining adequate drug concentration in plasma was vital for assessing the efficacy of drugs, monitor patient compliance, examine drug interactions and toxicity, determine interindividual variability in drug response, and tailor therapeutic regimen according to the patient's condition [4]. The growing use of immunosuppressive agents, particularly cyclosporine, tacrolimus, and sirolimus, also promoted the use of accurate TDM practice. Further, technological improvements in analytical methods for TDM happened. Mass spectrometry (MS) began to be recognized as a better tool when compared to traditional chromatography by offering improved sensitivity and specificity. From the 21st century, a new period of TDM began with pharmacogenetics supporting the theory of personalized medicine. Investigators progressively understood that genetic dissimilarities in drug-metabolizing enzymes influenced the drug responses. This led to the combination of genetic testing into conservative TDM protocols. Certain global initiatives aimed at regulating TDM practices have been launched by the International Federation of Clinical Chemistry (IFCC) and the Clinical and Laboratory Standards Institute (CLSI) [5]. Further, pharmacokinetic studies began for the Indian population. These helped establishing of region-specific TDM policies considering the diverse population of India. Despite all these efforts, the TDM practices are not that exhaustive in the Indian subcontinent, necessitating further improvement.

In this context, this narrative review aims to consolidate existing evidence regarding the development and current state of TDM in India. Particular emphasis is placed on its clinical applications, analytical techniques, implementation barriers, and emerging prospects. This review also seeks to identify gaps and recommend strategies to enhance and broaden TDM services throughout the Indian healthcare sector.

## Review

Need for TDM

The patient-related factors that TDM will be aiding the treatment process are the following.

Patients with certain comorbidities: Renal dysfunction lowers the removal of renally excreted drugs, such as lithium, which requires dosage adjustment. Apart from that, hepatic impairment influences the metabolism of several drugs such as valproate and carbamazepine [6]. Thus, TDM enables customised therapy, keeping in mind the associated comorbidities that the individuals are suffering from.

High-risk populations (e.g., pregnancy, pediatric patients, and elderly): Pregnant women undergo substantial pharmacokinetic deviations, with medications such as lamotrigine exhibiting about 50% increase in clearance negotiating its efficacy during pregnancy. In the pediatric population, drugs have various age-dependent pathways for managing drug disposition, whereas in older people, drugs undergo changed pharmacokinetics due to biological aging. Thus, TDM detects personalized variations in attributes of the drugs that might otherwise remain unknown, unless clinical consequences develop [7].

Suspected toxicity cases: TDM detects the drug concentration that underlies the clinical toxicity.

Monitoring medication adherence, especially in polypharmacy cases: Unpredictable low concentration of a drug usually indicates non-adherence. Moreover, patients taking multiple medications can have variability in drug concentration due to drug-drug interactions. For example, valproate increases lamotrigine concentrations by hindering its metabolism [8].

Difficulty in clinical assessment of the effects of the drugs: When drugs' therapeutic effect and associated toxicity cannot be differentiated easily from the underlying disease symptoms, measuring the plasma concentration of the drug offers insight into treatment.

Criteria for drugs for doing TDM

These are intrinsic properties of the drug or logistical system related consideration.

Narrow therapeutic window medications: Drugs such as lithium (therapeutic range: 0.8-1.2 mEq/L) and valproate (therapeutic range: 50-100 mcg/mL) need TDM due to their narrow therapeutic range. Drug concentration under those ranges may lead to ineffective treatment and, if above, can result in harmful effects.

Drugs with non-linear pharmacokinetics: For medications such as phenytoin, a minor rise in dosage yields vast concentration changes. In this type of non-linearity in pharmacokinetics, TDM delivers objective data to guide safe dose titration [9].

Drugs with a direct relationship between plasma levels and effects: TDM is of utmost importance when a consistent correlation exists between plasma concentrations and clinical outcomes of drugs such as aminoglycosides, allowing clinicians to target specific concentration ranges known to maximize benefits while minimizing toxicity risks.

Analytical techniques availability: Effective TDM necessitates dependable laboratory practices capable of precisely quantifying both parent drugs and related metabolites in biological samples. These criteria are particularly relevant in India, where ethnic heterogeneity, high burden of comorbidities, and variable access to specialized care increase the risk of suboptimal dosing and drug-related toxicity.

Procedure of TDM

**Figure 1:**
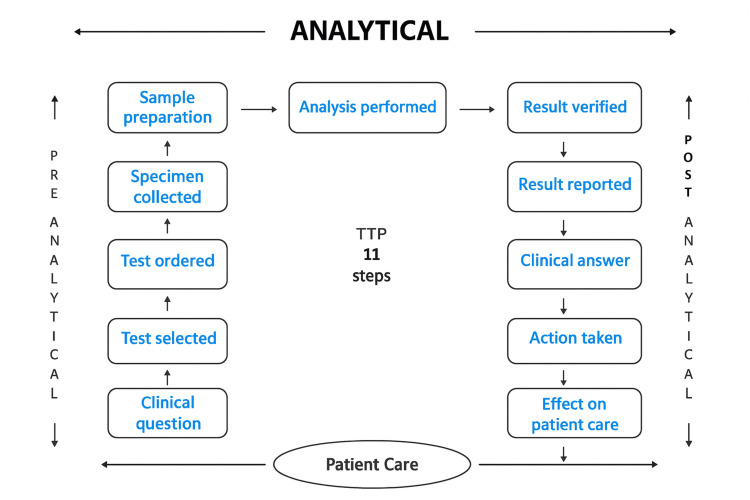
Schematic diagram projecting the 11-step total testing process (TTP) of TDM TDM: Therapeutic drug monitoring

The steps involved in TDM broadly fall under three main stages, as shown in Figure 1.

Pre-analytical Stage

**Figure 2:**
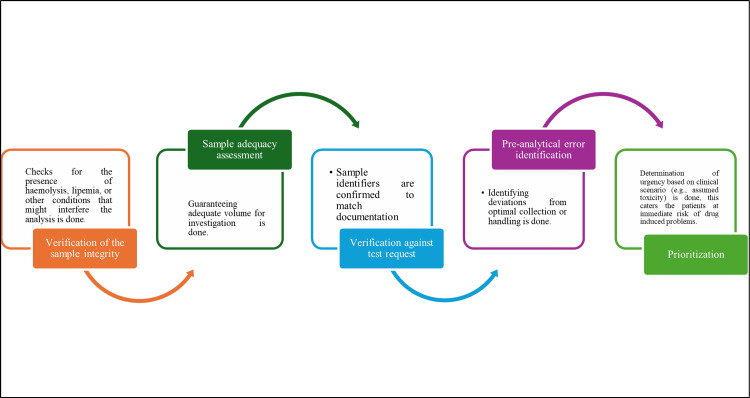
Schematic diagram of the sample receipt and assessment

It includes all steps that are done before the actual laboratory measurement, starting from the clinical decision of doing TDM to the collection and preparation of the samples. There are several factors affecting sample collection, its processing, handling, and triaging based on their urgent need [10] (Table 1, Figure 2).

**Table 1:** Pre-analytical factors influencing sample collection, handling, and processing in therapeutic drug monitoring Source: Ref [10]

Factors	Description
Factor affecting sample collection	
Timing precision	Sample collection time should be decided in relation to the timing of the last dose administered
Standardized techniques	Uniform collection procedures lessen unpredictability
Patient preparation	Fasting, posture, and physical activity of the patient may influence drug concentration levels
Sample stability considerations	Instantaneous processing or suitable preservation of the sample is to be done
Collection site	Collection from the site of drug administration must be prevented to avoid elevated concentration readings
Documentation requirements	Monitoring dosing history, concomitant medications and relevant clinical details will aid in interpreting the results
Factors affecting sample processing and handling	
Processing timeline	Immediate centrifugation must be performed to ensure sample stability
Preservation techniques	Some medications call for additional stabilizing agents to maintain their integrity
Aliquoting procedures	Keeps multiple samples ready, so comes in handy when repeat or further testing may be essential
Storage conditions	Suitable temperatures, light protection, and proper condition of the containers is needed to maintain the sample purity
Transport requirements	Timely delivery of the samples under appropriate storage conditions to the laboratory is of utmost importance, when the sample collection site and the laboratory are far away.

Analytical Stage

This stage is characterized by the principal laboratory testing process, where drug concentrations are analysed using specific methods. All the mentioned methodologies have different sensitivity and specificity; hence, selection is to be done wisely [11]. The brief description of the techniques [12-15] is presented in Table 2.

**Table 2:** A brief overview of the analytical methods used for TDM, outlining their principles, apparatus, specimen types, and reporting speed Source: Refs [12-15]

Sl. No.	Method	Principle	Apparatus	Sample	Reporting Time
Immunoassay Techniques					
1	Enzyme Immunoassay (EIA)	Uses enzyme-linked antibodies/antigens with colour change detection	Automated EIA analyser	Serum/plasma	Processing hundreds of samples daily
2	Fluorescence Polarization Immunoassay (FPIA)	Detects alterations in light polarization as fluorescent-labelled drugs interact with antibodies.	FPIA analyser	Serum/plasma	One to three minutes required per sample
3	Enzyme-Linked Immunosorbent Assay (ELISA)	Employs enzyme-tagged antibodies with substrate conversion for signal detection	Microplate reader, automated ELISA systems	Serum/plasma	Quick processing time
4	Chemiluminescent Immunoassay (CLIA)	Utilizes chemiluminescent reactions coupled with immune complex formation	CLIA analyser with photomultiplier	Serum/plasma	Sensitivity down to picogram levels
5	Enzyme-Multiplied Immunoassay (EMIT)	Competitive binding between enzyme-tagged drug analogues and sample drugs for antibody sites	EMIT analyser	Serum/plasma	Fast processing with little sample preparation
Chromatography Techniques					
6	Gas Chromatography (GC)	Division according to distribution between gaseous and liquid phases	GC instrument, often with MS detector	Serum/plasma	Highly specialized, slower process
7	High-Performance Liquid Chromatography (HPLC)	Separation based on distribution between two liquid phases	HPLC system	Serum/plasma/whole blood	Intermediate speed with batch handling
8	Ultra-High Performance Liquid Chromatography (UHPLC)	Improved separation using high-pressure techniques	UHPLC system	Serum/plasma/whole blood	Quicker than HPLC
9	Liquid Chromatography-Mass Spectrometry (LC-MS/MS)	Chromatographic analysis coupled with mass spectrometry detection	LC-MS/MS system	Serum/plasma/whole blood, low volume	Group processing
Other Techniques					
10	UV-Visible Spectrophotometry	Detects uptake of ultraviolet and visible light.	Spectrophotometer	Serum/plasma after extraction	Basic method with average speed
11	Atomic Absorption Spectroscopy (AAS)	Detects light absorption by isolated atoms	AAS instrument	Serum/plasma	Selective for metal-containing drugs
12	Ion Selective Electrodes (ISEs)	Direct detection of charged drug molecules.	ISE analyser	Whole blood/serum	Rapid, bedside testing

The following are the immunoassay techniques.

Enzyme immunoassay (EIA): It uses enzyme-linked antibodies or antigens with colorimetric detection systems. Modern automated EIA systems have excellent sensitivity and detection limits in the order of nanograms and permit the analysis of hundreds of samples in a day [12].

Fluorescence polarization immunoassay (FPIA): In this assay, drug molecules in the patient's sample compete with fluorescently labelled medications to bind with antibodies so that polarization measured varies inversely with drug levels in the sample. Thus, FPIA systems deliver quick results, highly suitable for emergency situations [16].

Enzyme-linked immunosorbent assay (ELISA): It is well-suited for the monitoring of monoclonal antibody therapies and anti-epileptic drugs, where it is useful in dose optimization through the differentiation between non-responders and responders. Microplate-based formats of ELISA allow high-throughput analysis and are appropriate for being used in high-volume clinical laboratories. Automated ELISA systems offer a good analytical performance, cost-effectiveness, and convenience for use in the routine clinical setting [17].

Chemiluminescent immunoassay (CLIA): It employs light-producing chemical reactions in relation to immunological binding. Thus, CLIA produces detection limits of the order of picograms and outperforms conventional techniques [18].

Enzyme-multiplied immunoassay (EMIT): When an enzyme-labelled drug combines with antibodies, its enzymatic activity gets suggestively reduced. EMIT enables rapid investigation with minimal sample preparation, making it ideal for high-volume clinical environments [19].

The following are the chromatographic methods.

Gas chromatography (GC): It offers advantages in quantifying volatile anesthetics, anticonvulsants, and certain psychotropic medications. When combined with mass spectrometry (GC-MS), the method offers better specificity. Its use has declined in modern days because of the presence of better methods [20].

High-performance liquid chromatography (HPLC): This technique helps in differentiating between active metabolites and parent drugs, which is crucial for drugs such as benzodiazepines and tricyclic antidepressants, where metabolites play a significant role in conferring the therapeutic effects. Current HPLC techniques in experimental laboratories frequently feature automated sample preparation, lowering the handling errors and increasing output [21].

Ultra-high performance liquid chromatography (UHPLC): Laboratory-grade UHPLC promotes faster interpretation times, which is vital for emergency situations such as those involving medications such as digoxin or lithium therapy. It also provides accurate differentiation of drugs from structurally related metabolites and endogenous substances. UHPLC's mixture of speed, sensitivity, and limited solvent consumption makes it suitable for high-volume TDM facilities [13].

Liquid chromatography-mass spectrometry (LC-MS/MS): Its capacity to measure distinct substances by separative chromatographic technique and classifying them through distinct mass fragmentation profiles makes it crucial for differentiating between structurally comparable drugs and metabolites. Overall, this method's sensitivity permits reliable recognition of medications even at low quantities, such as antipsychotics and targeted oncology treatments [14].

The following are the other methods used.

UV-visible spectrophotometry: It is used particularly in resource-constrained situations and for medications with comparatively simple analysis. It is employed to detect aminoglycosides and vancomycin, where detailed dosing within narrow therapeutic margins is vital to confirm efficacy while reducing the threat of nephrotoxicity and ototoxicity [15].

Atomic absorption spectroscopy (AAS): It precisely measures metal-based medications. It remains the preferred method for tracking lithium. The method also enables the assessment of crucial elements such as copper and zinc, which can moderate drug pharmacokinetics by affecting metabolic enzymes or transfer proteins [22].

Electrochemical technique: It uses the ion selective electrodes (ISEs), which carry out fast, direct recognition of ionized drug segments. Its ISE-based analyzers are capable of quantifying biologically active free ions where protein binding significantly impacts the drug pharmacokinetics. This helps clinicians prescribe appropriate dosing according to conditions such as hypoalbuminemia, pregnancy, etc. Haemolysis does not affect its interpretation. Current TDM applications often integrate ISEs together with chromatographic techniques, leveraging their fast detection rate and selectivity for ionic analytes [23].

In routine clinical practice, immunoassays are most appropriate for high-volume testing where rapid decision-making is required, such as emergency or inpatient settings. However, their susceptibility to cross-reactivity and limited ability to distinguish metabolites necessitate confirmation by chromatographic or mass-spectrometric methods in complex clinical scenarios.

Post-analytical Stage

This stage is about converting laboratory data into effective clinical information that directly impacts patient care through proper result clarification. It involves reporting drug concentrations along with suitable reference therapeutic ranges, providing clinical interpretation guiding the therapeutic management of the patient justified for distinct patient variables affecting pharmacokinetics, and introducing therapeutic actions such as modification in dose based on monitoring results [24].

TDM for generic medicines

TDM is equally important for generic and biosimilar drugs with a narrow therapeutic index, such as tacrolimus. There will be therapeutic failure with the minimal deviation in bioavailability, leading to possible transplant rejection or nephrotoxic damage. In addition, numerous agents (i.e., warfarin; risk of bleeding and thrombosis) and phenytoin (for seizure breakthrough or toxicity) pose similar dangers when switching to generic formulations. Regularly conducting TDM enables clinicians to identify and detect relevant variability in the individual's clinical response early and to appropriately modify dosages as needed to achieve optimal efficacy and safety [25,26].

TDM for different drugs in India

In India, TDM can be done for several drug categories, ranging from antimicrobials to several chemotherapy agents. Antiepileptics are one of the most monitored medications inthe Indian scenario. Increasing TDM practice for these drug categories necessitates the development of adequate, tailor-made assay protocols, standard operating procedures (SOPs), dedicated TDM education for physicians, and policy-level changes [27-32] (Table 3).

**Table 3:** A brief overview of the frequently monitored medications, including their therapeutic ranges, monitoring indications, and important clinical factors across different drug classes Source: Refs [27-32]

Drug Class	Drug Name	Therapeutic Range	Indications for TDM	Important Clinical Factors
Anticonvulsants	Phenytoin	10-20 μg/mL	Narrow therapeutic index, Saturable metabolism	Risk of breakthrough seizures and Concentration-related toxicity
	Carbamazepine	4-12 μg/mL	Autoinduction, Multiple drug interactions	Optimization of seizure control, Prevention of toxicity
	Valproic Acid	50-100 μg/mL	Variable protein binding	Managing effectiveness alongside hepatotoxicity and thrombocytopenia
	Lamotrigine	3.0-15.0 mcg/mL	Affected by enzyme inducers/inhibitors	Individualized dosing
Medications for Psychiatric Illness	Lithium	0.8-1.2 mmol/L	Narrow therapeutic window, Renal excretion	Bipolar illness control, Avoidance of kidney and central nervous system toxicity
	Tricyclic Antidepressants	100-300 ng/mL	Variable metabolism, Cardiotoxicity risk	Balancing therapeutic efficacy and minimizing anticholinergic effects
	Clozapine	350-600 ng/mL	Serious adverse effects	Surveillance for low white blood cell count
Drugs Acting on Cardiovascular and Respiratory System	Digoxin	0.8-2.0 ng/mL	Narrow therapeutic index, Variable pharmacokinetics	Treatment of cardiac failure and irregular heartbeat, especially critical for older adults
	Theophylline	5-15 µg/mL	Narrow therapeutic window	Bronchodilation in asthma and COPD
Immunosuppressants	Cyclosporine	100-300 ng/mL (varies by transplant type and time)	Narrow therapeutic window, Variable absorption	Managing rejection versus toxicity in transplant patients
	Tacrolimus	5-20 ng/mL	Significant inter-individual variability	Balancing graft acceptance with reduced toxicity
	Mycophenolate Mofetil	1–3.5 mg/L	Variable absorption and metabolism	Additional immunosuppressive treatment for transplantation
	Sirolimus/Everolimus	5-15 ng/mL	Narrow therapeutic window	Alternative or adjunct to calcineurin inhibitors
Antimicrobials	Vancomycin	15–20 mg/L	Nephrotoxicity risk	Used in gram positive endocarditis and osteomyelitis
	Rifampicin	8–24 mg/L	Inconsistent absorption, Enzyme induction	Additional immunosuppressive treatment for transplantation
	Voriconazole	1.0-4.0 μg/mL	Inherited polymorphisms affecting metabolism	Invasive fungal infections
Anticancer Drugs	Methotrexate	10-25 mg	Dose-dependent toxicity	High-dose therapy to guide leucovorin rescue
	Imatinib	>1000 ng/mL	Pharmacokinetic variability	Chronic granulocytic leukaemia, Stromal tumours of the GI tract
	5-Fluorouracil	2000-3000 µg/L	Narrow therapeutic window	Colon and rectal cancer treatment
Other Medications	Hydroxychloroquine	500 and 2000 ng/mL	Inconsistent drug distribution	Chronic inflammatory arthritis
	Acitretin	25-50 mg	Prolonged half-life	Psoriasis management

TDM in special populations

TDM becomes more important when it is about managing the pharmacotherapy in special patient populations who have varying pharmacokinetic and pharmacodynamic properties. These include children, the elderly, pregnant ladies, lactating mothers, individuals with altered hepatic or renal function, and critically ill patients. TDM is of paramount importance in pharmacotherapy for pediatric patients due to their uninterrupted growing composition, leading to changing pharmacokinetics with progressing age. Additionally, hepatic microsomal enzyme systems mature at varying rates, with several cytochrome P450 enzymes not reaching adult-level functionality until later in childhood; this too affects the pharmacokinetics of the drugs. These functional alterations make it difficult to precisely predict drug levels based only on weight-based dosing; thus, TDM will serve efficiently in this population. On the other hand, TDM also impacts the treatment process drastically in elderly patients. Age-associated functional changes include lowered total body water, lower serum albumin levels, an altered proportion of body fat, decreased hepatic perfusion, and advanced renal impairment. These variations can result in raised plasma concentrations of drugs, prolonged half-lives of drugs, higher free proportion of medications, and reduced clearance by the kidneys. Further, polypharmacy complicates drug therapy in the elderly [33]. Thus, TDM for certain drugs such as digoxin, lithium, and various anticonvulsants in this population assists clinicians in identifying age-related pharmacological shifts, facilitating suitable dose alterations to reduce toxicity while conserving therapeutic usefulness [34]. In pregnant ladies and lactating mothers, where altered pharmacokinetics are a concern, the practice of TDM becomes important. At the time of pregnancy, considerable physiological adaptations occur, such as alterations in protein binding due to large plasma volume, enhanced renal perfusion, altered hepatic enzyme activity, and reduced serum albumin levels. These variations typically lower the drug concentrations, which may weaken therapeutic efficacy. TDM for certain anticonvulsants, such as levetiracetam and lamotrigine, plays a significant role in maintaining seizure control while reducing risk to both the mother and fetus during the pregnancy. Similarly, tracking levels of antipsychotics and antimicrobials during pregnancy can also be done. Additionally, in lactating mothers, TDM helps in enhancing maternal treatment consequences and estimating the amount of drug exposure in the breastfeeding infant. Another category of patients requiring TDM is the critically ill patients. Problems such as hemodynamic instability can influence drug distribution and clearance, while systemic inflammation can alter protein binding and disrupt metabolic pathways. In such situations, TDM has become important, mostly for antimicrobial agents in situations such as sepsis, where reaching and sustaining adequate drug levels at the site of infection is vital for therapeutic success. Drugs such as vancomycin, beta-lactams, aminoglycosides, and antifungal agents are routinely monitored in intensive care facilities to tailor the treatment. Similarly, dealing with individuals with liver or kidney dysfunction requires careful consideration. Hepatic disorders decrease drug metabolism due to less enzymatic activity. TDM proves important in regulating medications, which are largely metabolized by the liver, such as benzodiazepines and certain antiepileptic drugs, preventing potential toxicity. In renal impairment, clearance of drugs along with their metabolites gets minimized, which necessitates dose individualization based on kidney function. Thus, TDM is regularly done for agents such as aminoglycosides, vancomycin, digoxin, and lithium, for which sensitivity changes in the uremic state [35].

Limitations of TDM

**Figure 3:**
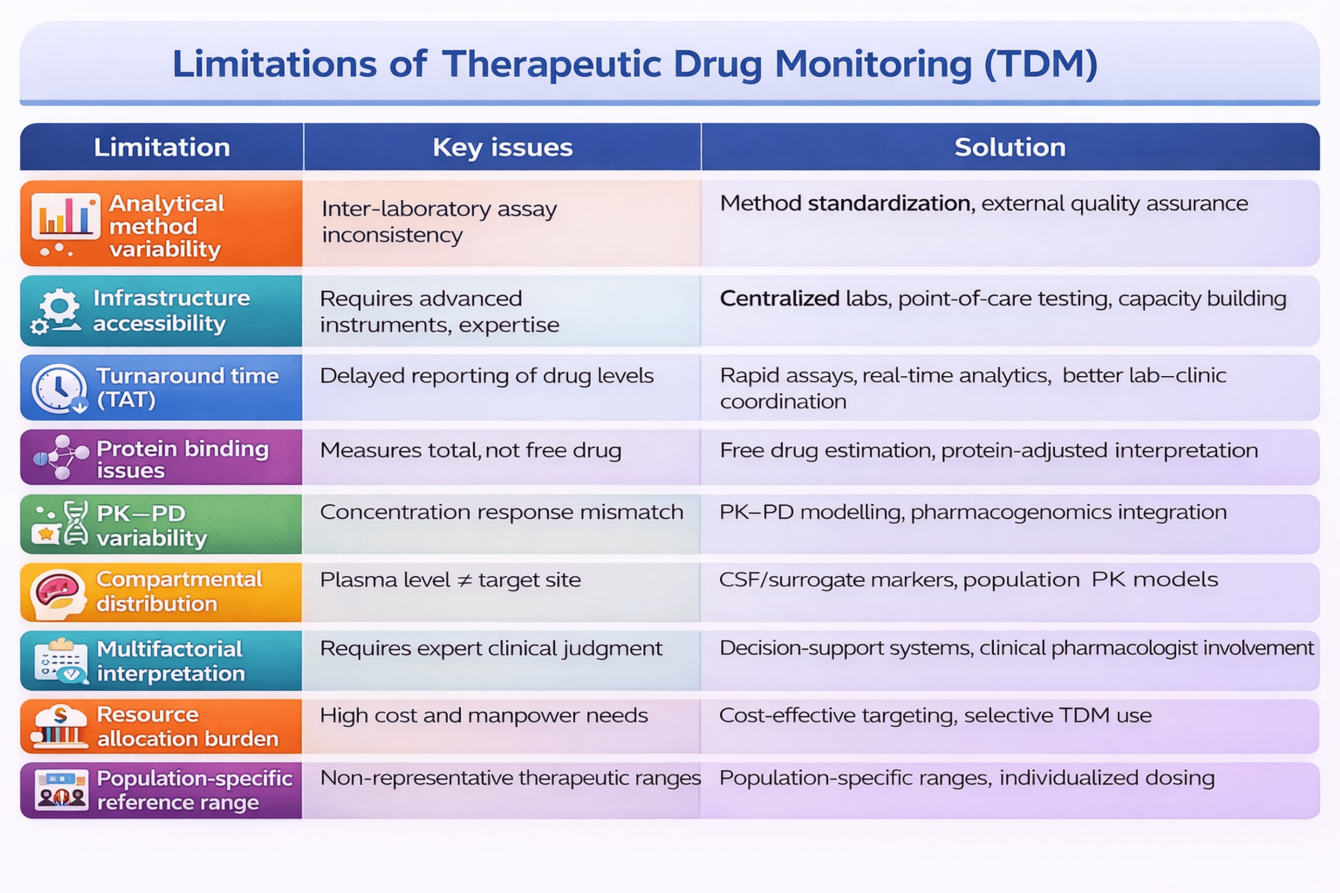
Limitations of TDM and their possible solutions Source: Ref [36]

There are several limitations pertaining to TDM practices described in Figure 3, along with their possible solution [36].

Emerging trends of TDM in India

In most of the tertiary centres in India, HPLC and LC-MS/MS are used as the gold standards for certain drug levels evaluation. These methods provide commendable precision and can differentiate between total and free drug concentrations, which is very crucial in patients with conditions such as hypoalbuminemia or in those individuals who are on a multi-drug regimen.

Point-of-Care and Decentralized Devices

Several fast-functioning, bedside technologies are coming up to counter the urban-rural testing discrepancy. These techniques are summarized in Table 4 [37-39].

**Table 4:** Emerging point-of-care techniques for therapeutic drug monitoring (TDM) practices Source: Ref [37-39]

Sl No.	Name of Device/Technique	Description	Advantages	Limitations
1	Colorimetric Paper-Based Assays	Uses F28TPP porphyrin reagents on dry slides that change to magenta colour based on drug (e.g., lithium) concentration. A digital/smartphone image under appropriate lighting helps quantify the levels.	Low-cost - Suitable for rural settings - Easy to use - Rapid testing	Affected by ambient lighting and camera resolution - Needs standardization
2	Optical Biosensors	Quinizarin-based fluorometric sensors detect emitted fluorescence after reacting with drug in saliva/sweat.	Non-invasive sampling - Rapid results	Short fluorescence emission lifetime - Signal interference from other ions in the sample
3	Electrochemical Biosensors	Use solid-contact ion-selective electrodes or screen-printed strips with 14-crown-4 ether to measure Lithium in whole blood through impedance changes.	No need for extensive preprocessing - Fast and specific	Potential cross-reactivity - Requires careful design
4	Microneedle Interstitial Fluid (ISF) Sensors	Painless microneedles (50–900 µm) pierce the skin to access ISF and monitor Lithium using impedance spectroscopy.	Continuous in vivo monitoring - Painless and minimally invasive	Difficulty in extracting sufficient ISF - Stability issues in biological environments
5	Lab-on-a-Chip (LOC) with Capillary Electrophoresis (CE)	Microfluidic systems that integrate sample prep and analysis. CE with conductivity detection enables lithium, Na⁺, K⁺ detection in small volumes.	Portable and compact - Multi-ion analysis in minimal volume - Suitable for field/rural use	Requires technical knowledge for operation - Cost of fabrication and miniaturization may be high

It is necessary to understand several factors to make TDM technologies feasible in rural India. These are the costs associated with each new technology, the ease of use for healthcare workers, and the turnaround time (TAT) needed to prepare samples for testing and reporting. Because of the lack of advanced laboratory facilities or adequately trained staff in many remote areas, TDM technologies that use small sample volume from, as well as those with automated reading capabilities (e.g., Rapidcard), will have a greater chance of being scaled up successfully. The diversity of testing conditions, environmental factors, and efficiency of technicians creates challenges for ensuring the reliability of results generated from testing that occurs at the outskirts.7

Point-of-care and decentralized devices face many obstacles in achieving both standardization and quality assurance, which still represents a major issue to be addressed. The analytical accuracy, precision, linearity, and robustness of the platforms must be comparable to the analytical methods currently employed in reference laboratories (i.e., HPLC or LC-MS/MS). It is therefore imperative to have a clear regulatory pathway established for validation, calibration, and post-market surveillance of the devices to assure both clinical safety and reproducibility of results. The lack of embedding technologies into national accreditation frameworks and external quality assurance programmes could lead to inconsistent dosing decisions and less-than-ideal patient outcomes when moving from research and development (R&D) to widespread utilisation. Therefore, the successful implementation of TDM in a decentralised manner in remote locations of rural India will require significant collaboration between regulatory bodies, academic institutions, and healthcare providers to achieve a balance of accessibility while maintaining the integrity of analytical processes and results.

Digital Health and Artificial Intelligence Integration

India stands at a pivotal point in expanding the scope and accessibility of highlighting the need for a unified vision toward personalized and precision-based therapeutics. The utilization of multiple digital health platforms and Al driven tools is recreating an entirely new era of TDM. Several digital tools may provide better scalable options to cope with the issues associated with accessibility to TDM. Several developments are already in progress for improving the TDM healthcare service. Some of those are described in Table 5 [40-43].

**Table 5:** Potential digital health services integration in the therapeutic drug monitoring (TDM) process Source: Refs [40-43]

Sl No.	Name	Description	Current Status in India
1	Mobile Health Applications (mHealth)	Mobile apps assist patients in adhering to medication schedules and follow-up appointments. They include features like automated reminders, real-time dose logging, user-friendly dashboards, and educational modules. Some are integrated with Bluetooth-enabled point-of-care devices for instant communication with healthcare providers.	Limited but growing use in urban areas; pilot projects for chronic disease monitoring exist. Integration with TDM-specific apps still under early development.
2	Electronic Medical Records (EMR)	EMRs with TDM dashboards and clinical decision support tools can detect when drug levels are out of therapeutic range, making real-time dose adjustment recommendations based on age, renal function, drug interactions, and labs. This improves clinical efficiency and reduces interpretation errors.	Widely implemented in tertiary care centres and select private hospitals; limited access in primary care. Integration of TDM-specific features is currently sparse.
3	AI-Based Dose Optimization Engines	AI-driven platforms use pharmacokinetic, clinical, and real-world datasets to tailor ideal dosing regimens. They continuously learn from patient responses and adjust for comorbidities and pharmacogenomic data when available, making them valuable for precision dosing.	Early research phase in India; academic trials in pharmacology departments. Commercial clinical adoption is yet to be realized on a large scale.
4	National Digital Health Infrastructure (NDHM)	NDHM and platforms like e-MANAS aim to integrate TDM with India's digital health records and services, ensuring real-time data exchange, unified patient records, and continuity of care across public and private settings.	Operational under Ayushman Bharat Digital Mission; integration of lab values including TDM is possible but not universally implemented across platforms.
5	Data Aggregation and Analytics Platforms	Centralized platforms collate TDM data from different health centres for nationwide monitoring. They help in evaluating quality indicators such as therapeutic range adherence, turnaround time, and adverse drug event trends. This can improve adherence, minimize drug-related failures, and standardize dosing protocols across the country.	Currently fragmented; large-scale integration and central registries for TDM are still in planning stages. Some disease-specific registries (like TB and HIV) are functional.

Future directions

The future of TDM in India depends on a convergence of the healthcare policy-based support, research, education of physicians, and systemic integration of SOPs in day-to-day practice. The eminent factors, which will further shape the TDM practice in India, are as follows.

Formation of India-specific guidelines: There is an immediate requirement to develop exclusive, India-centric TDM guidelines, especially for drug categories such as antiepileptics and mood stabilizers. These must be based on the already existing regional population data, pharmacogenetic variability, and present healthcare delivery standards of the country. Organizations such as the Indian Council of Medical Research (ICMR) or organizations under the National Mental Health Program (NMHP) can come forward and work in coordination with the tertiary teaching hospitals, providing TDM facilities to form a stringent TDM guideline specific to the Indian subcontinent [44].

Integration of TDM practice into national programs: Including TDM practice within the existing health frameworks, such as the NMHP, National Tuberculosis Elimination Programme (NTEP), and National Digital Health Mission, can further its institutionalization [45].

Education and capacity building: Formal inclusion of TDM-related modules in the curricula of MBBS (Bachelor of Medicine, Bachelor of Surgery) students, MD (Doctor of Medicine) students (especially in pharmacology and psychiatry residents), and M.Sc. (Medical Lab Technology) students will enhance competence. Nationwide and state-wise hands-on workshops on TDM and certain certification programs make the personnel well versed with TDM.

Infrastructure extension: Regarding regional laboratories with HPLC or LC-MS/MS, capabilities should be established through public-private partnerships (PPP). Mobile lab vans provided with basic TDM infrastructure can be a solution to serve remote peripheral areas. Public sector hospitals can work along with the National Accreditation Board for Testing and Calibration Laboratories (NABL)-accredited private labs to improve the TDM services. Purchasing point-of-care biosensors and incorporating them into primary health centres (PHCs) will further decentralize access to the TDM facility.

Research and innovation: India-specific pharmacokinetic and pharmacogenomic studies are crucial for improving TDM practices in India, especially forward-looking TDM trials providing information regarding the effect on the clinical outcomes and adherence with regular drug-level monitoring.

## Conclusions

The precise measurement of drug levels through TDM is a proven method for improving clinical outcomes in several diseases, such as bipolar disorder, epilepsy, fungal infections, and cancers, by maintaining drug levels within therapeutic ranges. In India, however, the full potential of TDM is not yet realized due to substantial costs of the tests, limited availability, and the lack of uniform national guidelines for TDM. Overcoming these challenges demands a comprehensive strategy, including integration of TDM into several nationwide health programmes; expansion of laboratory resources and point-of-care testing capabilities through partnerships between public and private sectors; empowerment of healthcare teams with the necessary knowledge and tools, such as advanced HPLC/LC-MS. Similarly, policy reforms, such as homogenizing laboratory qualification, guaranteeing coverage for TDM in health insurance, and fixing performance metrics, are essential for scaling it. Simultaneously, strategic research investments in forward-looking clinical trials and innovative biosensor technologies development will improve cost-effectiveness. Ultimately, with a unified effort amongst the regulatory bodies, clinicians, laboratory professionals, and patient advocates, the goal of achieving a future where all individuals receiving certain medications, such as anticancer drugs, immunosuppressants, antifungals, and neuropsychiatric drugs, will have access to precise and readily available monitoring services can be made possible. Thus, embedding TDM into India's healthcare system will reduce the adverse drug reactions associated with several narrow therapeutic range drugs, enhance the therapeutic outcomes, and make it a reality to enter a new era of individualized drug therapy.
